# Account of Diseases in an Eastern District of London, from February 20, to March 20, 1803

**Published:** 1803-04-01

**Authors:** 


### *Account of Diseases in an Eastern District of London, from February 20, to March 20, 1803.*

| | |
|---|---|
| ACUTE DISEASES. | |
| Synocha Catarrhalis | 47 |
| Pneumonia | 4 |
| Rheumatismus Acutus | 6 |
| Peripneumonia Notha | 7 |
| CHRONIC DISEASES. | |
| Phthisis Pulmonalis | 3 |
| Hæmoptoe | 4 |
| Hydrops Pectoris | 3 |
| Enterodynia | 7 |
| Hæmorrhagia Intestinalis | 2 |
| Dyspepsia | 12 |
| Angina | 4 |
| Menorrhagia | 5 |
| Fluor Albus | 7 |
| Amenorrhœa | 15 |
| Hysteria | 6 |
| Hypochondriasis | 2 |
| Paralysis | 2 |
| Rheumatismus Chronicus | 20 |
| PUERPERAL DISEASES. | |
| Peritonitis | 5 |
| Ephemera | 8 |
| Phlegmatia Dolens | 2 |
| Mastodynia | 10 |
| INFANTILE DISEASES. | |
| Varicella | 2 |
| Aphthæ | 4 |
| Herpes | 3 |
| Ophthalmia | 6 |
| Ophthalmia Purulenta | 3 |

The disease by which the greatest number of patients has lately been afflicted, and which has consequently engaged the principal attention of medical men, is a Catarrhal Fever. This disease has been so general as to claim the title of the reigning epidemic, and is very similar to one which prevailed a few years ago, and was denominated Influenza.

It has generally been introduced by chilliness and shiverings, which have been succeeded by violent pains in the head with some discharge from the eyes and nostrils, as in a common catarrh, together with hoarseness and cough. The pain in the head has in some cases been the first symptom, and has been succeeded by giddiness, sickness, and vomiting; and when these symptoms have in some measure subsided, the cough and difficulty of breathing have occurred. These symptoms have been observed in some instances to alternate, and as the cough and other affections of the chest have abated, the pain of the head and disorder of the stomach have been increased, and vice versa. The state of the bowels has been various, sometimes a diarrhœa has succeeded the nausea and sickness, and at other times an obstinate constipation has prevailed. In all cases there has been a considerable heat on the skin, a quick pulse, a white tongue, and a greater or less degree of thirst. The cough has sometimes been exceedingly violent, and has continued almost incessantly for several hours, whilst the sense of stricture on the chest and difficulty of breathing have been very distressing. The expectoration has in general been very free. Some patients have complained of pains in the limbs, very much resembling chronic rheumatism, and there have been instances in which the difficulty of breathing might be, in part, attributed to a similar affection of the intercostal muscles.

A number of anomalous symptoms have been combined with this disease; and, probably, the fatal effects which have been attributed to it, have in many instances arisen from another cause, particularly in old people, many of whom have fallen a sacrifice to Peripneumonia Notha, a disease which in many of its symptoms bears a resemblance to the present epidemic.

In the treatment of this disease much advantage has been derived from an early use of cathartics and emetics. If an antimonial has been combined with the cathartic, in such a manner as to occasion a gradually increasing nausea, and ultimately to produce vomiting; and if this has been succeeded by a copious evacuation of the bowels, the most important relief has been obtained; the pain of the head and uneasiness of the stomach have been greatly mitigated, if not entirely removed.

Bleeding has not in general been necessary, but we have seen some instances where the symptoms of Pneumonia have been so urgent, that an early bleeding would probably have afforded considerable relief. A blister to the sternum has been useful where Dyspnœa has prevailed. At the commencement of the disease, and during the continuance of febrile symptoms, saline remedies alone, or accompanied with antimonials, according to circumstances, are found to be a necessary part of the treatment. The different preparations of the squill are more useful at the decline of the disease than in the earlier stages of it. When febrile symptoms have abated, if the cough continues, the use of a gentle anodyne may be attended with advantage. Pulv. ipec. comp. has been found a convenient formula.

